# Cilostazol Ameliorates Motor Dysfunction and Schwann Cell Impairment in Streptozotocin-Induced Diabetic Rats

**DOI:** 10.3390/ijms25147847

**Published:** 2024-07-18

**Authors:** Lin-Li Chang, Yu-Ming Wu, Hung-Chen Wang, Kuang-Yi Tseng, Yi-Hsuan Wang, Yen-Mou Lu, Kuang-I Cheng

**Affiliations:** 1 Department of Microbiology and Immunology, Faculty of Medicine, College of Medicine, Kaohsiung Medical University, Kaohsiung 807378, Taiwan; elishawang1986@gmail.com; 2 Graduate Institute of Medicine, College of Medicine, Kaohsiung Medical University, Kaohsiung 807378, Taiwan; bear810206@yahoo.com.tw; 3 Department of Medical Research, Kaohsiung Medical University Hospital, Kaohsiung 807378, Taiwan; 4 Department of Neurosurgery, Chang Gung Memorial Hospital-Kaohsiung Medical Center, Chang Gung University College of Medicine, Kaohsiung 83301, Taiwan; m82whc@yahoo.com.tw; 5 Graduate Institute of Clinical Medicine, College of Medicine, Kaohsiung Medical University, Kaohsiung 807378, Taiwan; tsengkuangyi@gmail.com; 6 Department of Anesthesiology, Kaohsiung Medical University Hospital, Kaohsiung 807378, Taiwan; 7 Division of Pediatric and Spinal Orthopedics, Department of Orthopaedics, Kaohsiung Medical University Hospital, Kaohsiung 807378, Taiwan; yemoly@gmail.com

**Keywords:** diabetic rat, cilostazol, cholinergic neuron, schwannopathy, motor neuron, neuropathy

## Abstract

This study investigated the effects of cilostazol on motor dysfunction, spinal motor neuron abnormalities, and schwannopathy in rats with diabetes. Diabetes mellitus (DM) was induced in rats via femoral intravenous streptozotocin (STZ) injection (60 mg/kg). After successful DM induction, cilostazol was administered on day 15 via oral gavage (100 mg/kg/day) for 6 weeks until sacrifice. Behavioral assays, including motor function, were performed weekly. The sciatic nerve, L5 spinal cord, and spinal ventral root were collected to evaluate the expression of the glial fibrillary acidic protein (GFAP), myelin protein zero (P0), and choline acetyltransferase (ChAT) by immunofluorescence and Western blotting. DM rats displayed decreased running speeds, running distances, and toe spread but increased foot pressure. In addition, loss of non-myelinating Schwann cells and myelin sheaths was observed in the sciatic nerve and L5 spinal ventral root. Reduced numbers of motor neurons were also found in the L5 spinal ventral horn. Cilostazol administration significantly potentiated running speed and distance; increased hind paw toe spread; and decreased foot pressure. In the sciatic nerve and L5 spinal ventral root, cilostazol treatment significantly improved non-myelinated Schwann cells and increased myelin mass. ChAT expression in motor neurons in the spinal ventral horn was improved, but not significantly. Cilostazol administration may protect sensorimotor function in diabetic rats.

## 1. Introduction

Diabetes is a rapidly growing disease worldwide [1]. Hyperglycemia-induced diabetes is the primary cause of microvascular dysfunction and diabetic polyneuropathy (DPN). Microvascular pathology in human diabetic polyneuropathy includes vascular basement membrane thickening, capillary rarefaction, pericyte loss [2], endoneurial edema, and ischemia-induced fiber degeneration in tibial and sciatic nerves [3]. These vascular changes are strongly correlated with clinical defects and nerve pathology. There is strong evidence that nerve fiber degeneration and loss of nerve fibers are crucial in diabetic polyneuropathy [4,5]. Additionally, distal symmetrical sensorimotor polyneuropathy involving the motor system is most commonly seen, which includes motor weakness, an increased lower-limb amputation rate, and neuropathic pain [6]. 

Schwann cells are the main peripheral glial cells that maintain the peripheral nerve’s structure and wrap unmyelinated and myelinated axons. A complex interplay between Schwann cells, axons, and micro vessels during the development of diabetic neuropathy (DN) has been proposed [7]. Injury to Schwann cells causes schwannopathy, which affects both the vasculature and axons and is an integral factor in the pathogenesis of DN [7]. However, current knowledge about Schwann cells in response to diabetes is still insufficient, and treatment options for diabetes-induced peripheral neuropathy are still lacking. Motor deficits in humans with DN have been less researched than those in the sensory system, but they remain an important clinical complication. Motor neurons located within the ventral horn of the spinal cord, via the release of acetylcholine at the neuromuscular junction, are known to control skeletal muscles and a variety of motor behaviors. Choline acetyltransferase (ChAT) is the enzyme responsible for the biosynthesis of the neurotransmitter acetylcholine and can be used to identify motor neurons in the central and peripheral nervous systems. Data have shown that diabetes-related disorders of the cortico-muscular pathway exist, which explains the motor deficits in human diabetes [8].

Cilostazol, a potent inhibitor of phosphodiesterase (PDE)-III, increases cyclic adenosine monophosphate, leading to increases in blood flow, potent vasodilation, elevated high-density lipoprotein cholesterol, antiplatelet and anti-inflammatory activity, lowered serum triglycerides, and inhibited vascular smooth muscle cell growth [9]. In addition to being first marketed for the treatment of intermittent claudication [10], cilostazol has been investigated for other clinical uses, including peripheral arterial disease, cerebrovascular disease, the reduction of restenosis after coronary and peripheral endovascular interventions, and the prevention of secondary stroke [9,11]. In other studies, cilostazol was found to be somewhat effective in improving walking speeds and walking distances in patients with peripheral arterial disease [12,13]. Patients with critical limb ischemia receiving long-term cilostazol treatment had better primary patency, amputation-free survival, and a lower risk of mortality [14]. In contrast, a mini-review highlighted the benefits of cilostazol for treating diabetic nephropathy and retinopathy, indicating that cilostazol may not be effective in patients with diabetic peripheral neuropathy [15]. 

Diverse results of cilostazol in DN treatments had shown beneficial effects through the improvement of Na^+^/K^+^-ATPase activity by directly increasing cAMP and NO production in the human neuroblastoma cell line SH-SY5Y [16]. Furthermore, in experimental animal models of DN, a cilostazol-supplemented diet improved both nerve blood flow and nerve conduction [17]. Evidence from our previous DN rat experiments also indicated that cilostazol can blunt the responses to mechanical allodynia and has the potential to treat DN by attenuating sodium channels and glial cell dysregulation [18]. A report from Uehara found that cilostazol significantly improved motor nerve conduction velocity, restored nerve Na^+^/K^+^-ATPase activity, and decreased atrophic changes in the axons of the sural nerves of diabetic rats [19]. 

The impact of diabetes on sensory neurons during the development of peripheral neuropathy has been widely investigated [4]. However, studies regarding motor deficits and the cellular mechanisms of diabetic motor neuropathy in experimental animal models are limited [20,21]. Given the diverse and uncertain results of cilostazol in treating human diabetic neuropathy, further studies are necessary to confirm its benefits for this condition. This study aimed to investigate the cellular mechanisms underlying diabetes-induced motor defects and the effect of cilostazol on motor dysfunction in rats with STZ-induced DM.

## 2. Results

### 2.1. Cilostazol Ameliorated Neuropathic Pain

**Figure 1:**
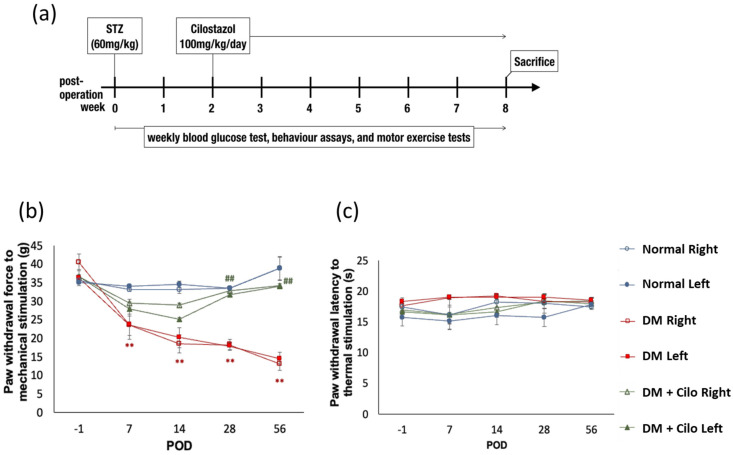
Analgesic effect of cilostazol (Cilo) in a diabetic (DM) rat model. (**a**) Experiment timelines outlining the relative time points of intravenous STZ (60 mg/kg) injection, the commencement of daily cilostazol (100 mg/kg) oral gavage, and sacrifice. Significant increases in tolerance against mechanical stimuli (**b**) and a mitigated blunt response to thermal hyperalgesia (**c**) in DM rats are indicated after cilostazol administration. Standard error (SE) are used to measure variability. Results are expressed as mean ± SE for a minimum of five rats for each group. Statistical significance was calculated with the Mann–Whitney U test. For normal versus DM groups, ** *p* < 0.01. For DM versus DM + cilostazol groups, ## *p* < 0.01. Abbreviation; POD: post-operative day, DM: diabetes mellitus.

Mechanical allodynia was observed on post-operative day 7 and persisted for 2 months in successfully induced diabetic rats (Figure 1b). The cilostazol-treated DM group (DM + cilo) showed a notable increase in tolerance to mechanical stimuli (Figure 1b). The levels of tolerance were sustained at levels similar to those in the control group and continued until the end of the 8th week. However, STZ-induced diabetic rats showed blunt responses to thermal stimuli and slightly increased, instead of decreased, thermal nociceptive thresholds (Figure 1c). Cilostazol administration seemed to mitigate the blunt response to thermal hyperalgesia and showed consistent thermal tolerance throughout the 8 weeks in the normal control group (Figure 1c). Overall, cilostazol ameliorated mechanical allodynia and tended to improve the blunt response to thermal hyperalgesia.

### 2.2. Cilostazol Improved Metabolic Flexibility

**Figure 2:**
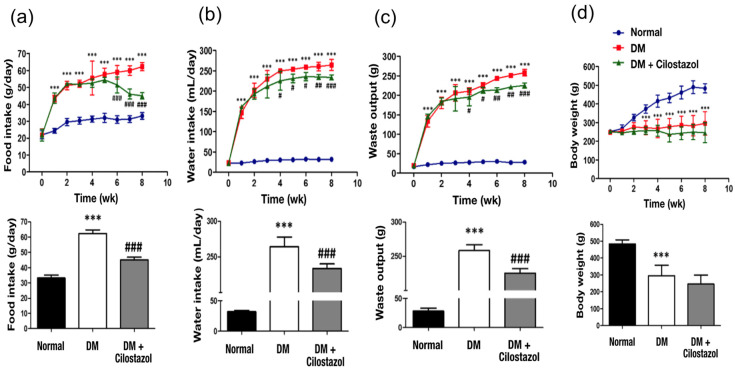
Effect of cilostazol on macronutrient factors in diabetic (DM) rats. Significantly decreased (**a**) food intake, (**b**) water intake, and (**c**) waste output are shown after cilostazol treatments. (**d**) Diabetes-induced body weight loss is not reversed following cilostazol administration. Standard error (SE) are used to measure variability. The data are shown as the mean ± SE. Statistical significance was calculated with a one-way ANOVA and Tukey’s HSD post hoc tests. For normal versus DM groups, *** *p* < 0.001. For DM versus DM + cilostazol groups, # *p* < 0.05; ## *p* < 0.01; ### *p* < 0.001. Abbreviation; wk: week, DM: diabetes mellitus.

The average daily consumption of food (Figure 2a), water (Figure 2b), and waste output (Figure 2c) was significantly higher in the DM group than in the normal group from day 7 post-STZ injection; this was maintained throughout the 8-week experiment. Following cilostazol administration, significantly lower food (Figure 2a), water (Figure 2b), and waste output (Figure 2c) were found compared to the DM group. However, body weight did not change significantly after cilostazol treatment (Figure 2d). 

**Figure 3:**
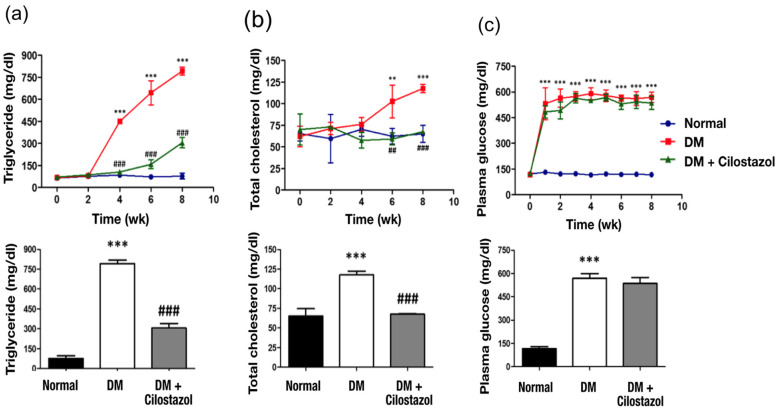
Effect of cilostazol on blood concentrations of triglycerides and cholesterol in diabetic (DM) rats. Significantly decreased blood levels of (**a**) triglycerides and (**b**) total cholesterol were observed after cilostazol treatment. (**c**) Diabetes-induced high blood glucose levels that were not reversed following cilostazol administration. Standard error (SE) are used to measure variability.The data are shown as the mean ± SE. Statistical significance was calculated using one-way ANOVA and Tukey’s HSD post hoc tests. For normal versus DM groups, ** *p* < 0.01; *** *p* < 0.001. For DM versus DM + cilostazol groups, ## *p* < 0.01; ### *p* < 0.001. Abbreviation; wk: week, DM: diabetes mellitus.

Four weeks after STZ injection, rats displayed abnormally high plasma triglyceride (Figure 3a) and total blood cholesterol levels (Figure 3b). Blood glucose levels gradually increased throughout the 2-month experimental period (Figure 3c). Except for blood glucose levels, significantly downregulated triglycerides and total blood cholesterol levels were observed after cilostazol treatment (Figure 3a–c).

### 2.3. Cilostazol Improved Motor Nerve Function

**Figure 4:**
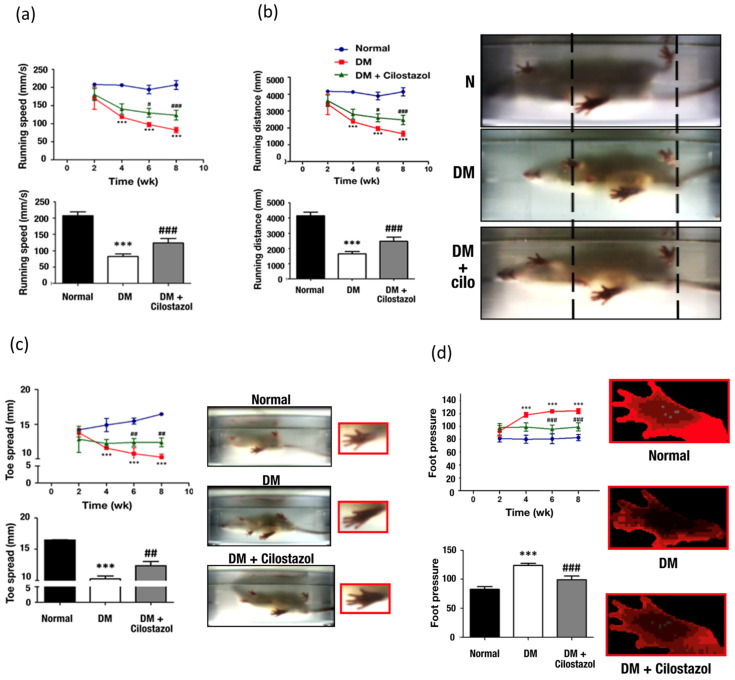
Effect of cilostazol on motor activity using the TreadScan software version 4 assay in diabetic (DM) rats. After cilostazol (cilo) treatment, (**a**) running speed, (**b**) running distance, and (**c**) toe spread were significantly increased. (**d**) Diabetes-induced higher foot pressure is reversed following cilostazol administration. Standard error (SE) are used to measure variability. The data are shown as the mean ± SE. Statistical significance was calculated using one-way ANOVA and Tukey’s HSD post hoc tests. For normal versus DM groups, *** *p* < 0.001. For DM versus DM + cilostazol groups, # *p* < 0.05; ## *p* < 0.01; ### *p* < 0.001. Abbreviation; wk: week, DM: diabetes mellitus, cilo: cilostazol.

To rule out the animal’s size and weight as significant confounding variables, the overall running speeds and distances calculated for each group were factored into the maximum strides and body lengths of each rat. For instance, the final running speed (mm/s) was calculated by normalizing the observed and recorded running speeds of each rat with their respective body weights and lengths. Four weeks after STZ injection, diabetic rats displayed significantly decreased running speed (Figure 4a), overall running distance (Figure 4b), toe spread distance (Figure 4c), and increased foot pressure (Figure 4d). Cilostazol administration significantly improved the running speed, overall running distance, toe spread, and foot pressure. Overall, this indicates the benefit of cilostazol for improving peripheral motor nerve function.

### 2.4. Cilostazol Renovates Myelin Sheath and Unmyelinated Schwann Cells in Peripheral Nerves

**Figure 5:**
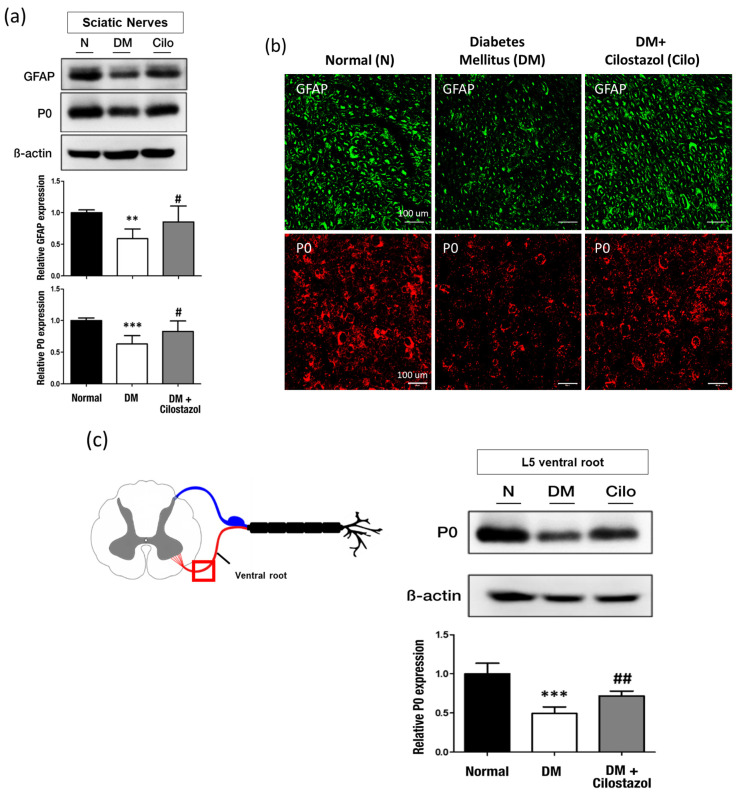
Effect of cilostazol (Cilo) on non-myelinated Schwann cells and myelin sheath expression in diabetic (DM) rats. (**a**) Glial fibrillary acidic protein (GFAP) and primary myelin protein (P0) expression in the sciatic nerve were analyzed by Western blotting. Quantification of Western blot data is shown on the side. (**b**) Immunofluorescence staining of GFAP- and P0-positive Schwann cells in the sciatic nerve. (**c**) P0 expression in the spinal ventral root was analyzed by Western blotting. Quantification of Western blot data is shown. Cilostazol has a significant effect on reversing diabetic-induced Schwann cell impairment. Standard error (SE) are used to measure variability. The data are shown as the mean ± SE. Scale bar: 100 μm; statistical significance was calculated with a one-way ANOVA and Tukey’s HSD post hoc tests. For normal versus DM groups, ** *p* < 0.01; *** *p* < 0.001. For DM versus DM + cilostazol groups, # *p* < 0.05; ## *p* < 0.01.

Hyperglycemia-induced schwannopathy affects both the vasculature and axons, which is an integral factor in the pathogenesis of DN. GFAP and P0 levels were used to measure schwannopathy. Diabetic rats showed significantly decreased GFAP and P0 expression in the sciatic nerve compared with the normal control group. After cilostazol administration, increased GFAP and P0 expressions were observed (Figure 5a,b). With respect to the ventral nerve root in the central spinal canal, there was a significant decrease in P0 expression in the L5 spinal ventral root of diabetic rats. This expression trend was reversed after cilostazol administration (Figure 5c). These results indicate that persistent hyperglycemia results in Schwann cells with thick myelination becoming thin or losing myelination, leading to myelinated nerve fiber damage; however, cilostazol improved diabetes-induced schwannopathy by preventing Schwann cell death and myelin fiber degeneration.

**Figure 6:**
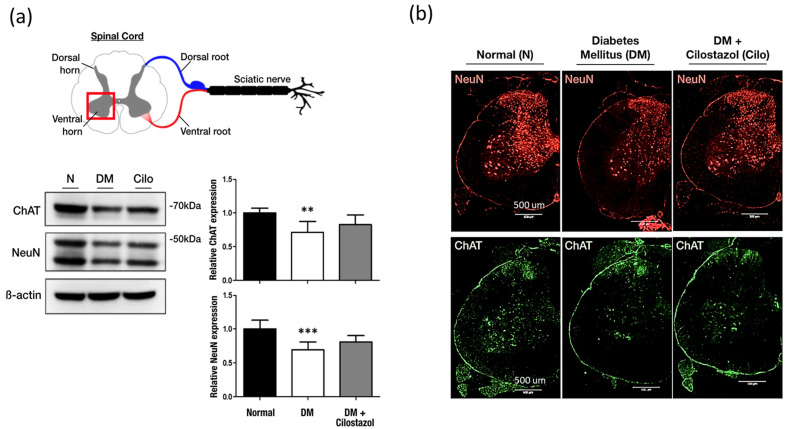
Effect of cilostazol on choline acetyltransferase (ChAT) expression in motor neurons in the spinal ventral horn in DM rats. (**a**) ChAT and NeuN (neuron cell marker) expression were analyzed by Western blotting. Quantification of Western blot data is shown on the side. (**b**) Immunofluorescence staining of ChAT and NeuN-positive neurons in the spinal ventral horn. A significant decrease in neuronal cells was observed when compared with normal controls. Cilostazol has little or no effect on ChAT motor neurons. Standard error (SE) are used to measure variability. The data are shown as the mean ± SE. Scale bar: 500 μm; statistical significance was calculated with a one-way ANOVA and Tukey’s HSD post hoc tests. For normal versus DM groups, ** *p* < 0.01; *** *p* < 0.001.

Significantly fewer ChAT-secreted motor neurons were observed in the L5 spinal ventral horn in diabetic rats compared to those in the normal control (Figure 6a,b). Although the cilostazol treatment group showed improved restoration of ChAT expression and neurons, there was no significant difference between diabetic rats with or without treatment (Figure 6a,b). This indicates that cilostazol can increase the expression of ChAT motor neurons but has no significant effect on them.

## 3. Discussion

The present study demonstrated that the daily administration of 100 mg/kg cilostazol for 6 weeks in diabetic rats significantly enhanced motor functional activities through increased running speeds and distances, expanded hind paw toe spread, and reduced diabetes-induced high foot pressure. Additionally, cilostazol treatment alleviated diabetes-induced mechanical hypersensitivity and led to a decrease in blood triglyceride and total cholesterol levels. Moreover, we observed a significant improvement in diabetes-induced demyelinating neuropathy in both the ventral nerve root and peripheral nerves, along with a restoration of the expressions of myelinated and non-myelinated Schwann cells in peripheral nerves. However, cilostazol treatment only partially reversed the reduced ChAT expression in motor neurons located in the spinal ventral horn, and this change was not statistically significant. Our results indicate that oral cilostazol effectively ameliorates diabetic neuropathy, particularly with respect to sensorimotor functional impairment. 

Diabetic peripheral neuropathy (DPN) is a complication predominantly resulting from microvascular damage, often combined with direct harm to Schwann cells in sensory or motor nerves [22]. Patients suffering from DPN are often observed with increased postural sway, prolong stance phase during walking [23], muscle mass reduction, and lower limb muscle weakness [6]. Our results mirrored these findings, observing that diabetic rats exhibited altered running patterns, characterized by reduced distance and speed, decreased hind paw toe spread, and higher foot pressure compared with the walking behaviors of normal rats. Furthermore, the present study demonstrated hyperglycemia-induced motor dysfunction, including demyelination of the ventral root and sciatic nerve, as well as reductions in the number of motor neurons in the ventral horn. These findings are in line with the observations reported by Souayah et al. that diabetic rats may display normal compound muscle action potential amplitude and latency while showing reductions in the number of motor units, axonal loss, as well as increased sprouting of nerve endings [24]. Moreover, persistent hyperglycemia reportedly could lead to rapid reductions in endoneurial blood flow within three days, followed by a decline in vascular function one week later, suggesting an association with decreases in motor nerve conduction velocity in the sciatic nerve in an experimental DPN rodent model [25]. This could be further corroborated by a recent article demonstrating the pronounced muscle fiber atrophy via a three-dimensional examination of structural and density abnormalities of muscular capillaries in a STZ-induced type-I DM mice model [26].

Diabetes-induced hypertriglyceridemia leads to a significant increase in free fatty acids, resulting in an overproduction of ROS in the mitochondria of macrovascular endothelial cells. This process is a substantial independent risk factor for diabetic peripheral neuropathy [27,28,29,30]. Clinical observations, epidemiologic evidence, and data from diabetic animal models all indicated that dyslipidemia adversely affected small unmyelinated axons and large fibers, resulting in axonopathy, schwannopathy, myelin degeneration, and ultimately lead to diabetic peripheral neuropathy [31,32,33,34]. In our current study, STZ-induced diabetic rats exhibited significant elevations in triglyceride and total cholesterol levels. This indicated not only persistent hyperglycemia but that hyperlipidemia induced chronic inflammation and contributes to increased reactive oxygen species (ROS) generation, impaired mitochondrial ATP production, decreased glyceraldehyde-3-phosphate dehydrogenase (GAPDH), and increased glycation of essential advanced-glycation end proteins (AGEs) and protein kinase C [30,33,35]. Among these hazard factors, glycolaldehyde, a precursor of AGEs, induces endoplasmic reticulum stress and apoptosis in Schwann cells that contribute to the pathogenesis of DN [36]. 

Sensorimotor peripheral neuropathy is a significant complication of diabetes mellitus. Although multiple factors contributed to DN, current therapies remain insufficiently effective. Alongside maintaining optimal glycemic control, oral medications such as tricyclic antidepressants, serotonin-noradrenaline reuptake inhibitors, anticonvulsants targeting calcium channels, and sodium channel blockers are recommended for pain mitigation [24,37]. Cilostazol, a specific cAMP phosphodiesterase inhibitor, used to recognize antiplatelet and vasodilator properties, has been demonstrated to improve motor function in diabetic rats through the amelioration of dysfunctional motor nerve conduction velocity, restoration of nerve Na^+^/K^+^-ATPase activity, and enhancement of mean myelinated fiber thickness, as well as via a reduction in axonal atrophic changes in peripheral nerves [17,19,38]. Additionally, cilostazol reduces plasma receptor for advanced-glycation end products [39], preserves mitochondrial function, decreases mitochondrial swelling, and decreases ROS production [40]. It exerts a positive effect on DN by enhancing nitric oxide production and elevating cellular cAMP levels [27]. Furthermore, cilostazol also enhances ornithine decarboxylase activity in the dorsal root ganglion and increases the rate of axonal regeneration in the sciatic nerve of DM rats [41]. Consequently, normalization of aberrant triglyceride and total blood cholesterol levels may also contribute to the restoration of motor function and the recovery of walking behaviors. 

Our previous findings indicated that cilostazol mitigated mechanical allodynia, attenuated sodium channel dysregulation, reduced microglia activation, and restored astrocytes in the spinal dorsal horn [18]. The current study further showed that cilostazol lowers triglyceride and total blood cholesterol levels and decreases the degeneration of Schwann cells in the peripheral nervous system. Notably, cilostazol significantly enhanced motor functions, which were measured via running speed, running distance, hind paw toe spread, and foot pressure, and ameliorated neuropathic pain. Therefore, oral cilostazol potentially offers diverse therapeutic effects to counteract hyperglycemia-induced diabetic complications. 

## 4. Materials and Methods

### 4.1. Studying Diabetes-Induced Animals

Eighty-five adult male Sprague Dawley rats weighing 250–300 g were used in this study. All rats were housed in plastic cages with soft bedding and kept under a 12 h light/dark cycle (light cycle, 7 a.m.–7 p.m.; dark cycle, 7 p.m.–7 a.m.) with access to food and water ad libitum. The rats were divided into three groups: normal control (Normal, n = 23), diabetic (DM, n = 31), and cilostazol-treated diabetic (DM + cilo, n = 31) groups. Type I diabetes was induced in rats by surgical exposure of the right femoral vein, followed by an intravenous injection of 60 mg/kg STZ (Sigma, St. Louis, MO, USA). Streptozotocin (STZ) is a cytotoxic glucose analogue and an alkylating agent that binds to pancreatic ß-cell GLUT-2 transporters, resulting in irreversible damage in the pancreas. When administered at a relatively high dosage, the chemical can cause massive pancreatic ß-cell destruction, leading to insulin deficiency, hyperglycemia, ß-cell necrosis, and weight loss within three days of application. The potency and relative ease of application of STZ presented the chemical as an economical way of inducing experimental DM models [42,43]. Successful induction of diabetes in injected rats was confirmed by the increase in random blood glucose levels to >500 mg/dL, which were assessed using an Accu-Chek^®^ Performa blood glucose assay kit (Roche, Taipei, Taiwan) on post-operative day (POD) two. Blood glucose levels and body weights were measured weekly. 

### 4.2. Cilostazol Administration

Cilostazol (tablet, OTSUKA PHARMACEUTICAL CO., LTD., Pfizer, Tokyo, Japan) was crushed and resuspended in ddH_2_O (50 mg/mL stock aliquots) and administered daily via oral gavage at 100 mg/kg according to our previous publication [18] from the second week of successful DM induction for up to 8 weeks until sacrifice (Figure 1a). 

### 4.3. Metabolic Function Analyses

The body weights and blood glucose levels of all rats were recorded weekly until termination. Blood lipid and total cholesterol values were assessed using the triglyceride assessment kit (ab65336, Abcam, Tokyo, Japan) and the cholesterol quantitation kit (MAK043, Merck, Rahway, NJ, USA). The total water and kibble consumption of the rats were recorded daily. Total excretion levels were estimated by subtracting the weight of each rat from the total weight of both the rat and cage contents at the end of a 24 h period. 

### 4.4. Behavioral Assays

Rats from all groups were subjected to mechanical allodynia and thermal hyperalgesia tests before and after STZ injections. Post-operation behavior assays were performed weekly until sacrifice. To examine mechanical allodynia and thermal hyperalgesia, the animals were acclimatized to the respective testing environments for up to 30 min. The testing facility for mechanical allodynia consisted of a metal mesh floor covered with a transparent plastic dome (8 × 8 × 18 cm). To measure hind paw withdrawal thresholds against mechanical stimuli, a dynamic plantar esthesiometer (UgoBasile, Gemonio, Italy) with an incremental increase of 2.5 g/s and a maximum cut-off threshold of 50 g was used. The withdrawal threshold of each paw was calculated as the average of four to six tests. To measure the latency of hind paw withdrawal from a thermal stimulus, each hind paw was set on a glass plate heated at 193 mW/cm^2^ by a directed infrared light beam through a 2 × 5 cm pinhole emitted from a moveable lightbox (UgoBasile Model 7370, UgoBasile, Gemonio, Italy). The thermal stimulus was terminated by the withdrawal of the paw from the glass plate or by automation at the 20 s cut-off time. The withdrawal threshold of each paw was calculated as the average of three to five continuous tests, with a minimum of 5 min of rest between each test. 

### 4.5. Motor Function Assays

Gait and foot pressure analyses were performed using the TreadScan software version 4 (TreadScan, CleverSys Inc., Reston, VA, USA). This CleverSys instrument provides a clear view of an animal’s paw prints for the analysis of motor deficits. In TreadScan systems, images are digitized and recorded using a built-in camera via BCam Capture version 2.00. In brief, the TreadScan system takes a video of an animal running on a transparent belt. A video of the underside view of the animal was obtained using a high-speed digital camera to capture and record the animal’s footprints as it walked or ran on the treadmill. Finally, the recorded data were analyzed using TreadScan-associated software.

### 4.6. Protein Extraction and Western Blotting

For protein extraction, freshly extracted L5 spinal cord, L5 ventral roots, and sciatic nerves were immediately frozen in liquid nitrogen and kept at −80 °C until further processing. Frozen samples were homogenized in a commercially available RIPA buffer (Invitrogen cat. 89901, USA), which contained a complete protease inhibitor mixture (Roche Diagnostics GmbH, Mannheim, Germany). For Western blotting, 50 µg of total protein from each sample was loaded onto a 10% sodium dodecyl sulfate-polyacrylamide gel and transferred to a polyvinylidene fluoride (PVDF) membrane (Millipore, Bedford, MA, USA). The membranes were hybridized with the following primary antibodies: β-actin (Millipore Sigma, MAB1501, Darmstadt, Germany), NeuN (Merck Millipore, MAB377, Burlington, MA, USA), choline acetyltransferase (Merck, AB144P, Rahway, NJ, USA), myelin protein zero (Abcam, ab31851, Waltham, MA, USA), and glial fibrillary acidic protein (Merck, ab5804, USA) for 24 h at 4 °C. This was followed by incubation with horseradish peroxidase-conjugated goat anti-mouse (Merck, AP124P, USA), goat anti-rabbit (Merck, AP132P, USA), and donkey anti-goat (Jackson ImmunoResearch, West Grove, PA, USA) secondary antibodies. Protein expression was visualized using enhanced chemiluminescence (ECL) detection reagents (Amersham Biosciences, Tokyo, Japan), captured, and processed using a UVP ChemiDoc-It^®^ 810 imager system (P/N 97–0645–05, 100–115 V~60 Hz, Cambridge CB4 1TG, UK). The expression levels were normalized to those of β-actin. The quantification was normalized to that of the normal control rats.

### 4.7. Histological Sample Preparation and Immunofluorescence 

The dissected L5 spinal cord and sciatic nerve tissues were fixed in 4% (*w*/*v*) paraformaldehyde and then saturated in 10–30% (*w*/*v*) sucrose in 0.02 mol/L PBS (pH 7.4). Once the samples were sufficiently dehydrated in 30% sucrose, they were embedded in OCT (FSC; FSC22 Clear; Surgipath; Leica Biosystems, Deer Park, IL, USA) in preparation for subsequent cryosectioning. L5 spinal cord samples were sectioned at 30 µm, and sciatic nerves were sectioned at 10 µm. The spinal cord samples were assayed for NeuN (Merck Millipore, MAB377, USA) and ChAT (Merck, AB144P, USA) expression in the ventral horn, whereas the sciatic nerve samples were assayed for GFAP (Abcam, AB53554, USA) and P0 (Abcam, ab31851) expression. After a minimum incubation period of 16 h, the samples were treated with fluorescence-conjugated Cy3 goat anti-rabbit (Merck, AP132C, USA), Alexa488 goat anti-mouse (Jackson ImmunoResearch, 115–545–003, USA), and Cy3 donkey anti-goat (Jackson ImmunoResearch, 765–165-147, USA) secondary antibodies for up to 2 h. Target protein expression was captured using a Zeiss LSM700 (Zeiss, Oberkochen, Germany) confocal microscope. 

### 4.8. Statistical Analyses

Group comparisons of behavioral responses were performed using the Mann–Whitney U test. The other tests were carried out with a one-way analysis of variance (ANOVA) and Tukey’s HSD post hoc tests using IBM^®^ SPSS^®^ Statistics 25 for statistical analyses. A *p*-value of ≤0.05 (α = 95%) was considered statistically significant.

## 5. Conclusions

In summary, from our study, oral cilostazol treatment preserves non-myelinated Schwann cells and prevents myelinated fiber degeneration tested from the sciatic nerve and L5 spinal ventral root in persistent hyperglycemic rats. Cilostazol administration protects diabetic rats from a sensorimotor dysfunctional state. However, hyperglycemia-induced schwannopathy is a key factor in the development of diabetic neuropathy. A further exploration is needed to clarify how cilostazol positively impacts schwannopathy.

Despite our best efforts, there are a lot of limitations to the current study. First and foremost, the DM model used in this project was limited to the relatively moderate length of eight weeks, whereas the majority of DPN related studies were carried out through a period of over two months. Lastly, this report prioritizes analyses and discussions regarding only the relative central and immediately adjacent PNS tissues associated with hindlimb mobilities and pain receptions. In order to validate these points, future studies could consider lengthening the experimental DM period to 4~6 months, which would also allow for later-stage treatment groups to test for the medication’s ability to moderate DPN in more advanced DM subjects. In addition, more distal PNS samples, such as tibial nerves, should also be included in future assessments to assess the impacts on distal PNS.

## Data Availability

The datasets generated during and/or analyzed during the current study are available upon request from the corresponding authors (m725006@kmu.edu.tw, kuaich@gmail.com).
